# Effects of Modulation of the Hedgehog and Notch Signaling Pathways on Osteoblast Differentiation Induced by Titanium with Nanotopography

**DOI:** 10.3390/jfb14020079

**Published:** 2023-01-30

**Authors:** Paola Gomes Souza, Leticia Faustino Adolpho, Helena Bacha Lopes, Denise Weffort, Alann Thaffarell Portilho Souza, Fabiola Singaretti Oliveira, Adalberto Luiz Rosa, Marcio Mateus Beloti

**Affiliations:** Bone Research Lab, School of Dentistry of Ribeirão Preto, University of São Paulo, Ribeirão Preto 14040-904, SP, Brazil

**Keywords:** hedgehog, nanotopography, notch, osteoblast, titanium

## Abstract

Background: The events of bone formation and osteoblast/titanium (Ti) interactions may be affected by Hedgehog and Notch signalling pathways. Herein, we investigated the effects of modulation of these signalling pathways on osteoblast differentiation caused by the nanostructured Ti (Ti-Nano) generated by H_2_SO_4_/H_2_O_2_. Methods: Osteoblasts from newborn rat calvariae were cultured on Ti-Control and Ti-Nano in the presence of the Hedgehog agonist purmorphamine or antagonist cyclopamine and of the Notch antagonist N-(3,5-Difluorophenacetyl)-L-alanyl]-S-phenylglycine t-butyl ester (DAPT) or agonist bexarotene. Osteoblast differentiation was evaluated by alkaline phosphatase activity and mineralization, and the expression of Hedgehog and Notch receptors was also evaluated. Results: In general, purmorphamine and DAPT increased while cyclopamine and bexarotene decreased osteoblast differentiation and regulated the receptor expression on both Ti surfaces, with more prominent effects on Ti-Nano. The purmorphamine and DAPT combination exhibited synergistic effects on osteoblast differentiation that was more intense on Ti-Nano. Conclusion: Our results indicated that the Hedgehog and Notch signalling pathways drive osteoblast/Ti interactions more intensely on nanotopography. We also demonstrated that combining Hedgehog activation with Notch inhibition exhibits synergistic effects on osteoblast differentiation, especially on Ti-Nano. The uncovering of these cellular mechanisms contributes to create strategies to control the process of osseointegration based on the development of nanostructured surfaces.

## 1. Introduction

Regenerative dentistry is based on developing strategies for inducting and maintaining cellular functions to promote oral tissue’s structural and functional reestablishment [1,2,3]. In this context, osteogenesis is characterized by the sequential steps of cell adhesion, proliferation, differentiation, extracellular matrix apposition, and mineralization dependent on cellular signalling pathways [4]. Among these pathways, the Hedgehog and Notch act in several cellular processes, including osteoblast differentiation [5,6].

The Hedgehog signalling pathway acts on cell proliferation during embryonic development, stem cell maintenance, tissue repair, and regeneration [7,8,9,10]. When the Hedgehog precursor protein binds to the patched-1 (PTCH1) receptor, the smoothened (SMO) protein becomes constitutively active. It induces the signalling cascade, stabilizing the family zinc finger 2 transcription factor (GLI2) [11]. GLI2 is translocated to the nucleus and induces transcription of target genes, such as *Ptch1*, *Gli1*, and *Gli2*, and bone morphogenetic proteins (BMPs) [12]. In the absence of Hedgehog-PTCH1 binding, the SMO protein remains inactive, and the suppressor of fused protein is activated, which downregulates the pathway and leads to the production of Gli3 [13]. Purmorphamine, an agonist of the Hedgehog pathway, increases the expression of the runt-related transcription factor 2 (Runx2) in osteoprogenitor cells, favours the differentiation of osteoblasts and the formation of mineralized extracellular matrix [14,15,16,17]. The effects of purmorphamine are induced by the stimulation of the signalling cascade and an increase in the expression of the genes *Gli1*, *Gli2*, *Ptch1*, and *Ptch2* [18,19]. Cyclopamine, an antagonist of the Hedgehog pathway, binds to the SMO protein and inhibits its activity by changing the protein conformation, inhibiting GLI1 and GLI2 [20,21]. Cyclopamine reduces the expression of Ptch1 and alters the pattern of the BMP-2 expression in osteoblasts [22,23].

The Notch signalling pathway is involved in cellular proliferation and differentiation processes, mainly during embryonic development [24,25]. The interaction of the ligand with the receptor releases the intracellular domain of the Notch receptor (NICD), which is translocated from the membrane to the nucleus, acting as a transcriptional coactivator. This domain heterodimerizes with a protein complex containing the DNA-binding protein, called recombination signal sequence-binding protein Jk. This interaction results in the removal of corepressors, recruitment of co-activators, and, consequently, in the transcription of target genes from the hairy/enhancer of split (*Hes*) and hairy-related transcription factor (*Hey*) families [26]. While the HES1 subtype favours, the HEY1 and HEY2 subtypes strongly inhibit RUNX2 activity [27,28,29,30,31]. Bexarotene is a Notch agonist that exerts its biological action by binding to the gamma-secretase protein complex, inducing activation of the pathway, which inhibits cell growth and differentiation [32]. In contrast, the Notch antagonist N-(3,5-Difluorophenacetyl)-L-alanyl]-S-phenylglycine t-butyl ester (DAPT), an inhibitor of the gamma-secretase protein complex, promotes osteogenesis [33,34].

The modulation of these cellular mechanisms using agonists and antagonists can affect the bone tissue and titanium (Ti) implants interaction, which also depends on surface characteristics. The mechanisms of favouring or inducing osteoblast differentiation by topographical and chemical characteristics of Ti surfaces have been widely discussed in the literature [35,36,37,38]. The investigation of the behaviour of cells cultured on Ti with nanotopography (Ti-Nano) obtained through chemical conditioning with H_2_SO_4_/H_2_O_2_ showed that nanotopography induces osteoblast differentiation in osteogenic and non-osteogenic environments [39,40,41]. To date, we have shown the participation of integrin, BMP, and Wnt signalling pathways in the osteogenic potential of this nanotopography [39,42,43,44,45,46]. As Hedgehog and Notch regulate osteogenesis and their possible involvement with the osteogenic potential of Ti-Nano has not been investigated yet, we hypothesized that Hedgehog and Notch signalling pathways participate in the nanotopography-induced osteoblast differentiation. Thus, this study aimed to investigate the effects of agonists and antagonists of these signalling pathways on the osteoblast differentiation of cells grown on Ti-Nano.

## 2. Materials and Methods

### 2.1. Ti Surface Modification and Characterization

All reagents were laboratory grade. There were two commercially pure grade Ti discs (13 × 2 mm, Realum, São Paulo, SP, Brazil) conditioned with a 10 N H_2_SO_4_ (Merck Millipore, Darmstadt, Hesse, Germany) and 30% H_2_O_2_ (Merck Millipore) solution for 4 h to create nanotopography (Ti-Nano), as previously described [40]. The control samples were non-treated Ti discs (Ti-Control). To characterize the surface topography, the Ti discs were examined under field emission scanning electron microscopy (SEM) operated at 5 kV (Inspect S50, FEI Company, Hillsboro, OR, USA).

### 2.2. Selection of the Concentrations of the Hedgehog and Notch Agonists and Antagonists

#### 2.2.1. Preparation of the Hedgehog and Notch Agonists and Antagonists

The Hedgehog agonist purmorphamine [2-(1-Naphthoxy)-6-(4-morpholinoanilino)-9-cyclohexylpurin] (Sigma-Aldrich, Saint Louis, MO, USA) was prepared at different concentrations of 0.5, 1, and 2 μM. The antagonist cyclopamine-KAAD [3-keto-N-aminoethyl-N’-aminocaproyldihydrocinnamoyl cyclopamine] (Calbiochem, Gibbstown, NJ, USA) was prepared at different concentrations of 10, 100, and 1000 nM. The Notch antagonist DAPT [GSI-IX, LY-374973, N-[N-(3,5-Difluorophenacetyl)-L-alanyl]-S-phenylglycine t-butyl ester] (Sigma-Aldrich) was prepared at different concentrations of 10, 15, and 20 μM, and the antagonist bexarotene [4-[1-(5,6,7,8-Tetrahydro-3,5,5,8,8-pentamethyl-2-naphthalenyl)ethenyl)benzoic acid] (Santa Cruz Biotechnology, Dallas, TX, USA) was prepared at different concentrations of 0.1, 0.5, and 1 μM. All agonists and antagonists were dissolved in the vehicle dimethylsulfoxide (DMSO, Sigma-Aldrich) and diluted in a culture medium, and the concentrations were selected based on the literature [18,19,32,34].

#### 2.2.2. Isolation and Culture of Osteoblasts

After approval of the Ethics Committee on the Use of Animals of the School of Dentistry of Ribeirão Preto, University of São Paulo (Protocol # 2019.5.309.58.0), the osteoblasts were isolated from calvariae of newborn male Sprague-Dawley rats aged 2–4 days, as previously described [47,48]. Osteoblasts (2 × 10^4^ cells per well) were cultured in a minimum essential medium, alpha modification (α-MEM, (Gibco-Life Technologies, Waltham, MA, USA)) supplemented with 10% foetal bovine serum (Gibco-Life Technologies), 5 μg/mL ascorbic acid (Gibco-Life Technologies), 7 mM β-glycerophosphate (Sigma-Aldrich), and 50 μg/mL gentamicin in 24-well polystyrene culture plates (Corning Life Sciences, Corning, NY, USA). The cultures were kept for up to 17 days at 37 °C in a humidified atmosphere (5% CO_2_ and 95% atmospheric air) in the presence of either the vehicle (DMSO), the Hedgehog, or Notch agonists and antagonists.

#### 2.2.3. Analysis of Gene Expression by Real-Time Polymerase Chain Reaction (RT-qPCR)

The osteoblastic marker osteopontin (*Opn*) gene expression was evaluated on day 10 by RT-qPCR. The total RNA was extracted using the SV Total RNA Isolation System kit (Promega, Fitchburg, WI, USA) and reverse transcription reaction was carried out to synthesize the complementary DNA (cDNA) using the High-Capacity cDNA Reverse Transcription Kit (Thermo Fisher Scientific, Waltham, MA, USA). For RT-qPCR, the SYBR Green system, and primers (Table 1) were used in the QuantStudio™ 7 Flex System device (Applied Biosystems, Waltham, MA, USA). The reactions were done (n = 4) and the data analysed using the cycle threshold value (Ct). The expression of the constitutive gene eukaryotic translation initiation factor 2B, subunit 1 alpha (*Eif2b1*) was evaluated, and the 2^-ddCt^ method was used to compare the gene expression of the experimental groups [49,50].

#### 2.2.4. Analysis of the ALP Activity by Fast Red Staining

The ALP activity was evaluated on day 7 using Fast red staining, as previously described [51]. The cultures were incubated with 1 mL of a solution containing 1.8 mM Fast Red-TR Salt (Sigma-Aldrich), 0.9 mM Naphthol AS-MX phosphate (Sigma-Aldrich), and 4 mg/mL dimethylformamide (Sigma-Aldrich) for 30 min. The wells were dried and macroscopic images of the whole wells with the stained cultures were obtained with a high-resolution camera (Canon EOS Digital Rebel Camera, Canon, Lake Success, NY, USA). The stained areas of the whole wells were quantified by counting the pixels using the ImageJ 1.52 software (National Institute of Mental Health, Bethesda, MD, USA). The data (n = 5) were expressed as a percentage of area.

#### 2.2.5. Analysis of the Extracellular MATRIX Mineralization by Alizarin Red Staining

The formation of a mineralized extracellular matrix was evaluated on day 17 by Alizarin red staining. The cultures were fixed with 10% formalin at 4 °C for 24 h, dehydrated with alcohol, dried, and stained with 2% Alizarin red (Sigma-Aldrich), and the quantification was performed according to what was previously described [52]. The absorbance was measured in a spectrophotometer (BioTek Instruments Inc., Winooski, VT, USA) using a wavelength of 405 nm, and the data (n = 5) were expressed as absorbance.

### 2.3. Effects of the Hedgehog Signalling Modulation on Osteoblast Differentiation and Expression of Hedgehog Receptors in Cells Grown on Ti-Control and Ti-Nano

The osteoblasts were cultured on Ti-Control and Ti-Nano discs in 24-well polystyrene culture plates (Corning Life Sciences) at a density of 2 × 10^4^ cells per disc in the presence of either vehicle, the agonist purmorphamine (Sigma-Aldrich) or the antagonist cyclopamine (Calbiochem) at the previously selected concentrations. The analyses of the gene expression of *Runx2*, *Opn*, *Alp*, *Gli1*, *Gli2*, and *Gli3* using the primers presented in Table 1, ALP activity, and extracellular matrix mineralization were performed as already detailed here, at the same time points. Additionally, the expression of RUNX2 and GLI1 proteins was evaluated by western blot on day 10.

#### Analysis of the Protein Expression by Western Blot

Western blot detected the expression of RUNX2 and GLI1 proteins on day 10 as previously described [40]. The cells were lysed and 25 μg of the total protein was denatured, separated in SDS polyacrylamide electrophoresis gel, and transferred to a PVDF membrane (Bio-Rad Laboratories, Hercules, CA, USA). The antibodies used were primary antibody either anti-RUNX2 (8486, 1:1000; Cell Signaling Technology, Danvers, MA, USA), anti-GLI1 (ab273018-1:1000; Abcam, Cambridge, UK) or anti-GAPDH (sc-25778, 1:1000; Santa Cruz Biotechnology, Dallas, TX, USA), and secondary antibody goat anti-rabbit IgG (7074, 1:3000, Cell Signaling Technology). The proteins were revealed with Clarity^TM^ Western ECL Substrate (PerkinElmer Life Sciences, Waltham, MA, USA), and the images were obtained in a G: BOX device (Syngene, Cambridge, UK). The RUNX2 and GLI1 expressions were quantified (n = 3) using ImageJ Software (NIH, Bethesda, MD, USA) and normalized to GAPDH.

### 2.4. Effects of the Notch Signalling Modulation on Osteoblast Differentiation and the Expression of Notch Receptors in Cells Grown on Ti-Control and Ti-Nano

Osteoblasts were cultured on Ti-Control and Ti-Nano discs in 24-well polystyrene culture plates (Corning Life Sciences) at a density of 2 × 10^4^ cells per disc in the presence of either vehicle, the antagonist DAPT (Sigma-Aldrich) or the agonist bexarotene (Santa Cruz Biotechnology) at the previously selected concentrations. The analyses of the gene expression of *Runx2*, *Opn*, *Alp*, *Hes1*, *Hey2*, and *Hey3* using the primers presented in Table 1, the protein expression of RUNX2 and HES1, ALP activity, and extracellular matrix mineralization were done as already detailed here, at the same time points. The antibody used to detect HES1 by western blot was anti-HES1 (11,988, 1:1000; Cell Signalling Technology), and the secondary antibody was goat anti-rabbit IgG (7074, 1:3000, Cell Signaling Technology).

### 2.5. Effects of the Combination of the Hedgehog and Notch Signalling Modulation on the Gene Expression of Bone Markers in Cells Grown on Ti-Control and Ti-Nano

To evaluate the effects of combining the modulation of both Hedgehog and Notch signalling, osteoblasts were cultured on Ti-Control and Ti-Nano discs in 24-well polystyrene culture plates (Corning Life Sciences) at a density of 2 × 10^4^ cells per disc in the presence of either vehicle, the association of the Hedgehog agonist purmorphamine (Sigma-Aldrich) with the Notch antagonist DAPT (Sigma-Aldrich) or the association of the Hedgehog antagonist cyclopamine (Calbiochem) with the Notch agonist bexarotene (Santa Cruz Biotechnology) at the previously selected concentrations. The analyses of the gene expression of *Runx2*, *Opn*, and *Alp* using the primers presented in Table 1 were done, as already detailed here, at the same time.

### 2.6. Statistical Analysis

The software SigmaPlot free trial version 15.0 (Systat Software Inc., San Jose, CA, USA) was used to analyse the data. The data of concentrations’ selection of the Hedgehog and Notch agonists and antagonists were analysed by one-way ANOVA, followed by Tukey’s post-test. The data of the effects of the Hedgehog and Notch signalling modulation on osteoblast differentiation and the expression of Hedgehog and Notch receptors in the cells grown on Ti-Control and Ti-Nano were analysed using two-way ANOVA, followed by the Tukey’s post-test. The results were expressed as the mean ± standard deviation (SD), and the significance level was established at *p* ≤ 0.05.

## 3. Results

### 3.1. Ti-Control and Ti-Nano Surfaces

**Figure 1:**
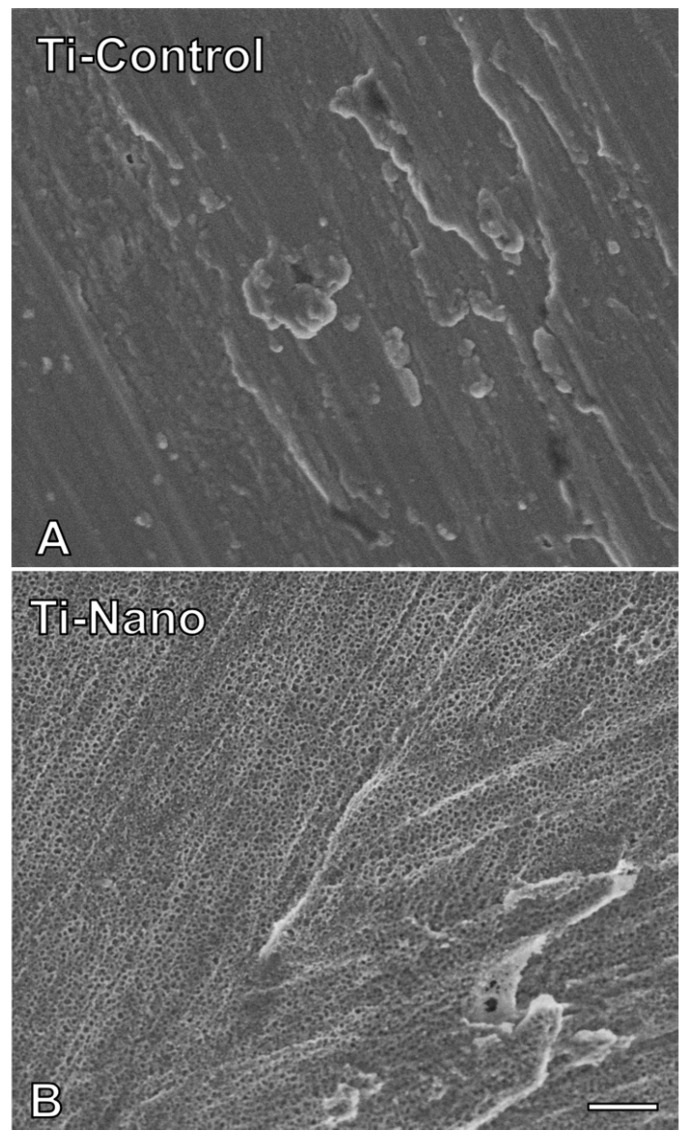
Surface topography of the titanium (Ti) discs. Images generated by scanning electron micrography of polished Ti ((**A**), Ti-Control) and nanostructured Ti ((**B**), Ti-Nano). Scale bar (**A**,**B**): 200 nm. Original magnification: 100,000×.

The SEM demonstrated that Ti-Control presents a polished surface (Figure 1A), and the Ti-Nano produced by H_2_SO_4_/H_2_O_2_ treatment exhibited nanopores over the entire surface (Figure 1B).

### 3.2. Selection of the Concentration of the Hedgehog and Notch Agonists and Antagonists

**Figure 2:**
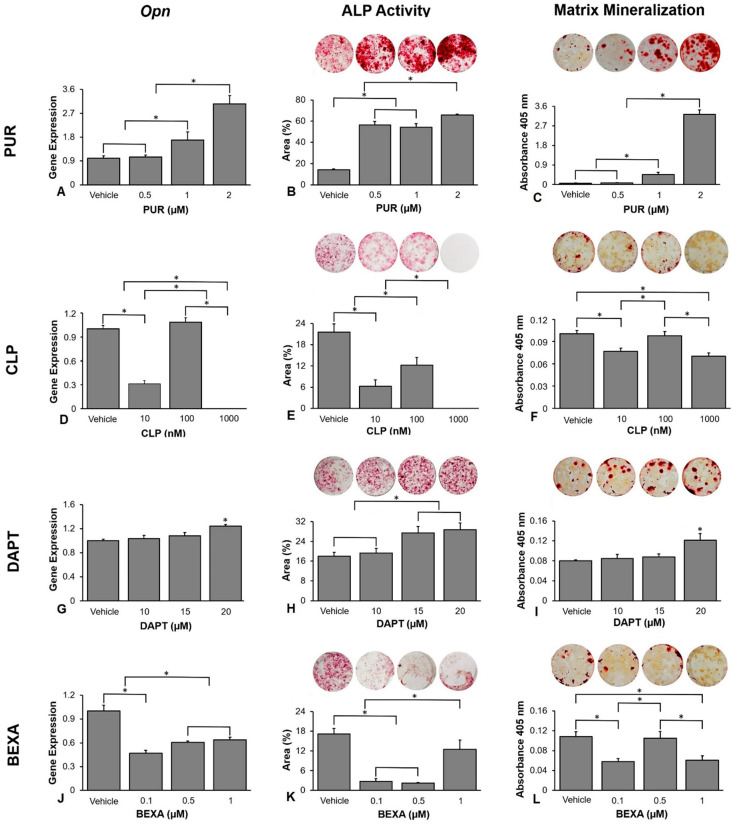
Selection of the concentrations of the Hedgehog and Notch agonists and antagonists based on their effects on osteoblast differentiation. Osteopontin (*Opn*) gene expression on day 10 (**A**), alkaline phosphatase (ALP) activity on day 7 (**B**), and extracellular matrix mineralization on day 17 (**C**) in osteoblasts cultured on polystyrene with either vehicle or the Hedgehog agonist Purmorphamine (PUR) at the concentrations of 0.5, 1, and 2 μM of (PUR). *Opn* gene expression on day 10 (**D**), alkaline phosphatase (ALP) activity on day 7 (**E**), and extracellular matrix mineralization on day 17 (**F**) in osteoblasts cultured on polystyrene with either vehicle or with cyclopamine (CLP) at the concentrations of 10, 100, and 1000 nM. *Opn* gene expression on day 10 (**G**), alkaline phosphatase (ALP) activity on day 7 (**H**), and extracellular matrix mineralization on day 17 (**I**) in osteoblasts cultured on polystyrene with either vehicle or with DAPT at the concentrations of 10, 15, and 20 μM. *Opn* gene expression on day 10 (**J**), alkaline phosphatase (ALP) activity on day 7 (**K**), and extracellular matrix mineralization on day 17 (**L**) in osteoblasts cultured on polystyrene with either vehicle or with bexarotene (BEXA) at the concentrations of 0.1, 0.5, and 1 μM. The original diameter of the bottom of the polystyrene wells presented in (**B**,**C**,**E**,**F**,**H**,**I**,**K**,**L**) is 15.62 mm. The data are presented as the mean ± SD, and the asterisks (*) indicate a statistically significant difference (*p* ≤ 0.05).

The selection of the concentration of the Hedgehog and Notch agonists and antagonists was based on the *Opn* gene expression, ALP activity, and extracellular matrix mineralization of cells grown on polystyrene. Higher osteoblast differentiation induced by purmorphamine was observed at a concentration of 2 M (Figure 2A–C). The *Opn* gene expression was higher at a concentration of 2 μM compared with 1 μM (*p* < 0.001), which was higher than 0.5 μM (*p* < 0.001) and vehicle (*p* < 0.001), and there was no statistically significant difference between 0.5 μM and vehicle (*p* = 0.992, Figure 2A). The ALP activity was higher at the concentration of 2 μM compared with 1 μM (*p* < 0.001), 0.5 μM (*p* < 0.001), and vehicle (*p* < 0.001), which was lower in vehicle compared with 0.5 μM (*p* < 0.001) and 1 μM (*p* < 0.001), without statistically significant difference between them (*p* = 0.520, Figure 2B). The extracellular matrix mineralization was greater at the concentration of 2 μM compared with 1 μM (*p* < 0.001), which was higher than 0.5 μM (*p* < 0.001) and vehicle (*p* < 0.001), and there was no statistically significant difference between the vehicle and 0.5 μM (*p* = 1.000, Figure 2C). The best effect of cyclopamine in decreasing osteoblast differentiation was observed at the concentration of 1000 nM (Figure 2D–F); however, as cyclopamine 1000 nM exhibited some cytotoxic effect, we selected 10 nM for further experiments. The *Opn* gene expression was lower at the concentration of 1000 nM compared with 10 nM (*p* < 0.001), which was lower than 100 nM (*p* < 0.001) and vehicle (*p* < 0.001), and there was no statistically significant difference between them (*p* = 0.549, Figure 2D). The ALP activity was lower at the concentration of 1000 nM compared with 10 nM (*p* = 0.001), which was lower than 100 nM (*p* < 0.001) that was lower than the vehicle (*p* < 0.001, Figure 2E). The extracellular matrix mineralization was lower at the concentrations of 1000 nM and 10 nM compared with 100 nM (*p* = 0.002 and *p* = 0.009) and vehicle (*p* = 0.001 and *p* = 0.004), without statistically significant differences between 1000 nm and 10 nM (*p* = 0.553), and 100 nM and vehicle (*p* = 0.937, Figure 2F). The best effect of DAPT in increasing osteoblast differentiation was observed at the concentration of 20 μM (Figure 2G–I). The *Opn* gene expression was higher at the concentration of 20 μM compared with 15 μM (*p* = 0.028), 10 μM (*p* = 0.005), and vehicle (*p* = 0.002), and there were no statistically significant differences among vehicle, 10 μM and 15 μM ((*p* = 0.384, *p* = 0.775, and *p* = 0.894, Figure 2G). The ALP activity was higher at the concentrations of 20 μM and 15 μM compared with 10 μM (*p* < 0.001 and *p* < 0.001) and vehicle (*p* < 0.001 and *p* < 0.001), without statistically significant differences between 20 μM and 15 μM (*p* = 0.831), and 10 μM and vehicle (*p* = 0.871, Figure 2H). The extracellular matrix mineralization was higher at the concentration of 20 μM compared with 15 μM (*p* = 0.041), 10 μM (*p* = 0.027), and vehicle (*p* = 0.014), and there were no statistically significant differences among vehicle, 10 μM and 15 μM (*p* = 0.866, *p* = 0.990, and *p* = 0.964, Figure 2I). The best effect of bexarotene in decreasing osteoblast differentiation was observed at the concentration of 0.1 μM (Figure 2J–L). The *Opn* gene expression was lower at 0.1 μM compared with 0.5 μM (*p* = 0.013) and 1 μM (*p* = 0.003), which were lower than vehicle (*p* < 0.001 and *p* < 0.001), and there was no statistically significant difference between 0.5 μM and 1 μM (*p* = 0.809, Figure 2J). The ALP activity was lower at the concentrations of 0.1 μM and 0.5 μM compared with 1 μM (*p* < 0.001, *p* < 0.001), which was lower than vehicle (*p* = 0.007), and there was no statistically significant difference between 0.1 μM and 0.5 μM (*p* = 0.918, Figure 2K). The extracellular matrix mineralization was lower at the concentrations of 0.1 μM and 1 μM compared with 0.5 μM (*p* = 0.024 and *p* = 0.034) and vehicle (*p* = 0.012 and *p* = 0.016), without statistically significant differences between 0.1 μM and 1 μM (*p* = 0.994), and 0.5 μM and vehicle (*p* = 0.948, Figure 2L). Based on these results, we selected the following concentrations for the further experiments of this study: purmorphamine 2 μM, cyclopamine 10 nM, DAPT 20 μM, and bexarotene 0.1 μM.

### 3.3. Effects of the Hedgehog Signalling Modulation on Osteoblast Differentiation and the Expression of Hedgehog Receptors in Cells Grown on Ti-Control and Ti-Nano

**Figure 3:**
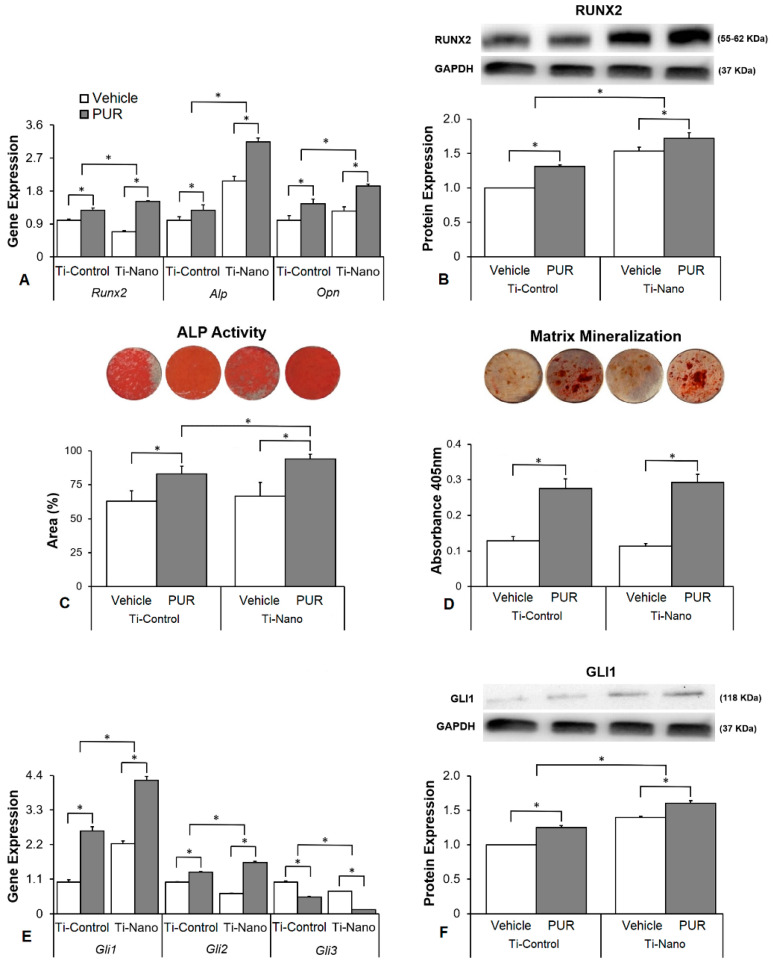
Effects of the Hedgehog agonist purmorphamine (PUR) on osteoblast differentiation and the expression of Hedgehog receptors in osteoblasts grown on polished Ti (Ti-Control) and Ti with nanotopography (Ti-Nano). The gene expression of the osteoblastic markers runt-related transcription factor 2 (*Runx2*), osteopontin (*Opn*), and alkaline phosphatase activity (*Alp*) on day 10 (**A**), RUNX2 protein expression on day 10 (**B**), ALP activity on day 7 (**C**), extracellular matrix mineralization on day 17 (**D**), gene expression of the Hedgehog receptors zinc finger 1, 2 and 3 transcription factors (*Gli1*, *Gli2*, and *Gli3*) on day 10 (**E**) and GLI1 protein expression on day 10 (**F**) in osteoblasts cultured on Ti-Control and Ti-Nano with either vehicle or PUR 2 μM. The original diameter of the Ti discs presented in C and D is 13 mm. The data of gene expression (n = 4), protein expression (n = 3), ALP activity (n = 5), and extracellular matrix mineralization (n = 5) are presented as mean ± SD, and * indicate statistically significant differences (*p* ≤ 0.05).

The interaction between Ti surfaces and purmorphamine treatment affected the gene expression of *Runx2* (*p* ≤ 0.001), *Alp* (*p* ≤ 0.001), and *Opn* (*p* = 0.043, Figure 3A). Purmorphamine upregulated the gene expression of *Runx2* (*p* < 0.001 and *p* < 0.001), *Alp* (*p* = 0.001 and *p* = 0.004), and *Opn* (*p* < 0.001and *p* < 0.001) in cells grown on both Ti-Control and Ti-Nano (Figure 3A). In the presence of vehicle, the gene expression of *Runx2* (*p* < 0.001) was lower, while *Alp* (*p* < 0.001) and *Opn* (*p* = 0.008) was higher in cells grown on Ti-Nano than on Ti-Control (Figure 3A). In the presence of purmorphamine, the gene expression of *Runx2* (*p* < 0.001), *Alp* (*p* < 0.001), and *Opn* (*p* < 0.001) was higher in cells grown on Ti-Nano than on Ti-Control (Figure 3A). The interaction between Ti surfaces and purmorphamine treatment also affected the RUNX2 protein expression (*p* ≤ 0.001), Figure 3B). Purmorphamine increased the RUNX2 protein expression (*p* = 0.003 and *p* = 0.016) in the cells grown on both Ti-Control and Ti-Nano (Figure 3B). In the presence of vehicle or purmorphamine, the RUNX2 protein expression (*p* < 0.001 and *p* < 0.001) was higher in cells grown on Ti-Nano than on Ti-Control (Figure 3B). The interaction between Ti surfaces and purmorphamine treatment did not affect the expression of ALP activity (*p* = 0.270, Figure 3C). Purmorphamine increased the ALP activity (*p* < 0.001 and *p* < 0.001) in cells grown on both Ti-Control and Ti-Nano (Figure 3C). In the presence of vehicle, the ALP activity (*p* = 0.431) was not affected by Ti surfaces (Figure 3C). In the presence of purmorphamine, the ALP activity (0.028) was higher on Ti-Nano than Ti-Control (Figure 3C). The interaction between Ti surfaces and purmorphamine treatment did not affect the extracellular matrix mineralization (*p* = 0.111, Figure 3D). Purmorphamine increased the extracellular matrix mineralization (*p* < 0.001 and *p* < 0.001) in the cells grown on both Ti-Control and Ti-Nano (Figure 3D). In the presence of vehicle or purmorphamine, the extracellular matrix mineralization (*p* = 0.284 and *p* = 0.221) was not affected by Ti surfaces (Figure 3D). The interaction between Ti surfaces and purmorphamine treatment affected the expression of *Gli1* (*p* ≤ 0.001), *Gli2* (*p* ≤ 0.001), and *Gli3* (*p* ≤ 0.001, Figure 3E). Purmorphamine upregulated the gene expression of *Gli1* (*p* < 0.001 and *p* < 0.001), *Gli2* (*p* < 0.001 and *p* < 0.001), and downregulated *Gli3* (*p* < 0.001and *p* < 0.001) in cells grown on both Ti-Control and Ti-Nano (Figure 3E). In the presence of vehicle, the gene expression of *Gli1* (*p* = 0.018) was higher, while *Gli2* (*p* < 0.001) and *Gli3* (*p* < 0.001) were lower in cells grown on Ti-Nano than on Ti-Control (Figure 3E). In the presence of purmorphamine, the gene expression of *Gli1* (*p* = 0.001) and *Gli2* (*p* < 0.001) was higher, while *Gli3* (*p* < 0.001) was lower in cells grown on Ti-Nano than on Ti-Control (Figure 3E). The interaction between Ti surfaces and purmorphamine treatment also affected the GLI1 protein expression (*p* < 0.001, Figure 3F). Purmorphamine increased the GLI1 protein expression (*p* = 0.003 and *p* = 0.016) in cells grown on Ti-Control and Ti-Nano (Figure 3F). In the presence of vehicle or purmorphamine, the GLI1 protein expression (*p* < 0.001 and *p* < 0.001) was higher in cells grown on Ti-Nano than on Ti-Control (Figure 3F).

**Figure 4:**
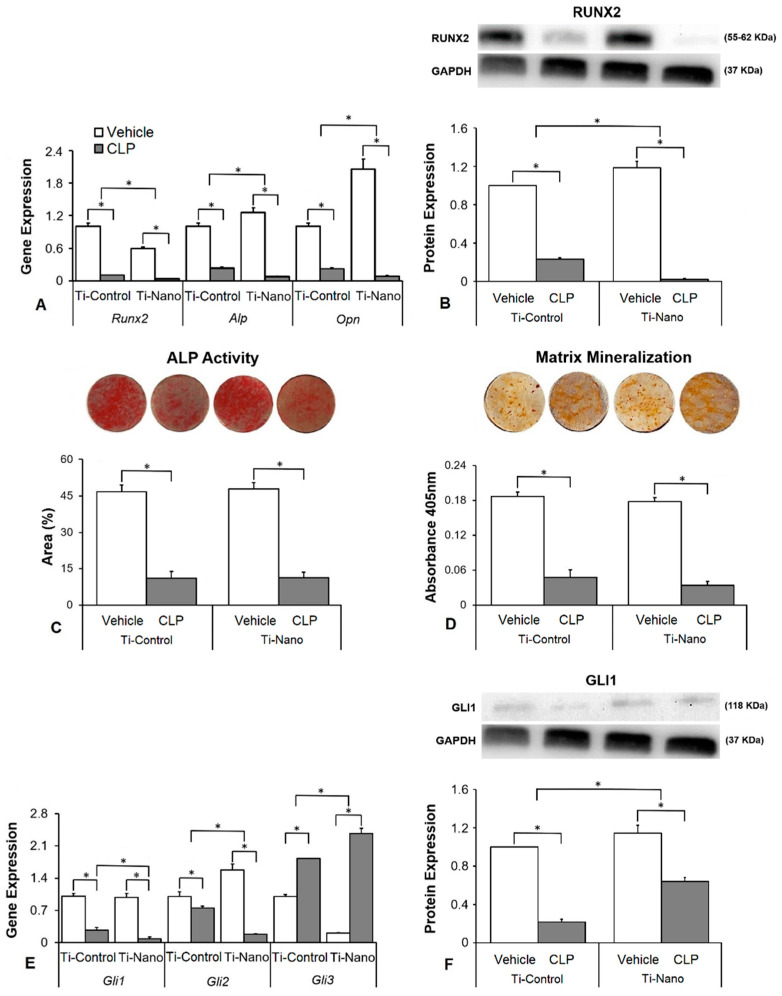
Effects of the Hedgehog antagonist cyclopamine (CLP) on osteoblast differentiation and the expression of Hedgehog receptors in osteoblasts grown on polished Ti (Ti-Control) and Ti with nanotopography (Ti-Nano). The gene expression of the osteoblastic markers runt-related transcription factor 2 (*Runx2*), osteopontin (*Opn*), and alkaline phosphatase activity (*Alp*) on day 10 (**A**), RUNX2 protein expression on day 10 (**B**), ALP activity on day 7 (**C**), extracellular matrix mineralization on day 17 (**D**), gene expression of the Hedgehog receptors zinc finger 1, 2 and 3 transcription factors (*Gli1*, *Gli2*, and *Gli3*) on day 10 (**E**) and GLI1 protein expression on day 10 (**F**) in osteoblasts cultured on Ti-Control and Ti-Nano with either vehicle or CLP 10 nM. The original diameter of the Ti discs presented in C and D is 13 mm. The data of gene expression (n = 4), protein expression (n = 3), ALP activity (n = 5), and extracellular matrix mineralization (n = 5) are presented as the mean ± SD, and * indicates statistically significant differences (*p* ≤ 0.05).

The interaction between Ti surfaces and cyclopamine treatment affected the gene expression of *Runx2* (*p* ≤ 0.001), *Alp* (*p* = 0.001), and *Opn* (*p* ≤ 0.001, Figure 4A). Cyclopamine downregulated the gene expression of *Runx2* (*p* < 0.001 and *p* < 0.001), *Alp* (*p* < 0.001 and *p* < 0.001), and *Opn* (*p* < 0.001 and *p* < 0.001) in cells grown on both Ti-Control and Ti-Nano (Figure 3B). In the presence of vehicle, the gene expression of *Runx2* (*p* < 0.001) was lower, while *Alp* (*p* = 0.020) and *Opn* (*p* < 0.001) were higher in cells grown on Ti-Nano than on Ti-Control (Figure 4A). In the presence of cyclopamine, the gene expression of *Runx2* (*p* = 0.038), *Alp* (*p* = 0.009), and *Opn* (*p* < 0.001) was lower in cells grown on Ti-Nano than on Ti-Control (Figure 4A). The interaction between Ti surfaces and cyclopamine treatment also affected the RUNX2 protein expression ((*p* ≤ 0.001), Figure 4B). Cyclopamine decreased the RUNX2 protein expression (*p* < 0.001 and *p* < 0.001) in cells grown on both Ti-Control and Ti-Nano (Figure 4B). In the presence of vehicle, the RUNX2 protein expression (*p* < 0.001 and *p* < 0.001) was higher, while in the presence of cyclopamine, it was lower in cells grown on Ti-Nano than on Ti-Control (Figure 4B). The interaction between Ti surfaces and cyclopamine treatment did not affect the ALP activity (*p* = 0.647, Figure 4C) and extracellular matrix mineralization (*p* = 0.653, Figure 4D). Cyclopamine decreased the ALP activity (*p* < 0.001 and *p* < 0.001, Figure 4C) and extracellular matrix mineralization (*p* < 0.001 and *p* < 0.001, Figure 4D) in cells grown on both Ti-Control and Ti-Nano. In the presence of vehicle or cyclopamine, the ALP activity (*p* = 0.513 and *p* = 0.993, Figure 4C) and extracellular matrix mineralization (*p* = 0.185 and *p* = 0.059, Figure 4D) were not affected by Ti surfaces. The interaction between Ti surfaces and cyclopamine treatment affected the expression of *Gli1* (*p* ≤ 0.001), *Gli2* (*p* ≤ 0.001), and *Gli3* (*p* = 0.001, Figure 4E). Cyclopamine downregulated the gene expression of *Gli1* (*p* < 0.001 and *p* < 0.001) and *Gli2* (*p* < 0.001 and *p* < 0.001) and upregulated *Gli3* (*p* < 0.001 and *p* < 0.001) in cells grown on both Ti-Control and Ti-Nano (Figure 4E). In the presence of vehicle, the gene expression of *Gli1* (*p* = 0.346) was not affected, while *Gli2* (*p* < 0.001) was higher and *Gli3* (*p* < 0.001) was lower in cells grown on Ti-Nano than on Ti-Control (Figure 4E). In the presence of cyclopamine, the gene expression of *Gli1* (*p* = 0.006) and *Gli2* (*p* < 0.001) was lower, while *Gli3* (*p* < 0.001) was higher in cells grown on Ti-Nano than on Ti-Control (Figure 4E). The interaction between Ti surfaces and cyclopamine treatment also affected the GLI1 protein expression (*p* ≤ 0.001, Figure 4F). Cyclopamine decreased the GLI1 protein expression (*p* < 0.001, *p* < 0.001) in cells grown on both Ti-Control and Ti-Nano (Figure 4F). In the presence of vehicle or cyclopamine, the GLI1 protein expression (*p* = 0.006 and *p* < 0.001) was higher in cells grown on Ti-Nano than on Ti-Control (Figure 4F).

### 3.4. Effects of the Notch Signalling Modulation on Osteoblast Differentiation and the Expression of Notch Receptors in the Cells Grown on Ti-Control and Ti-Nano

**Figure 5:**
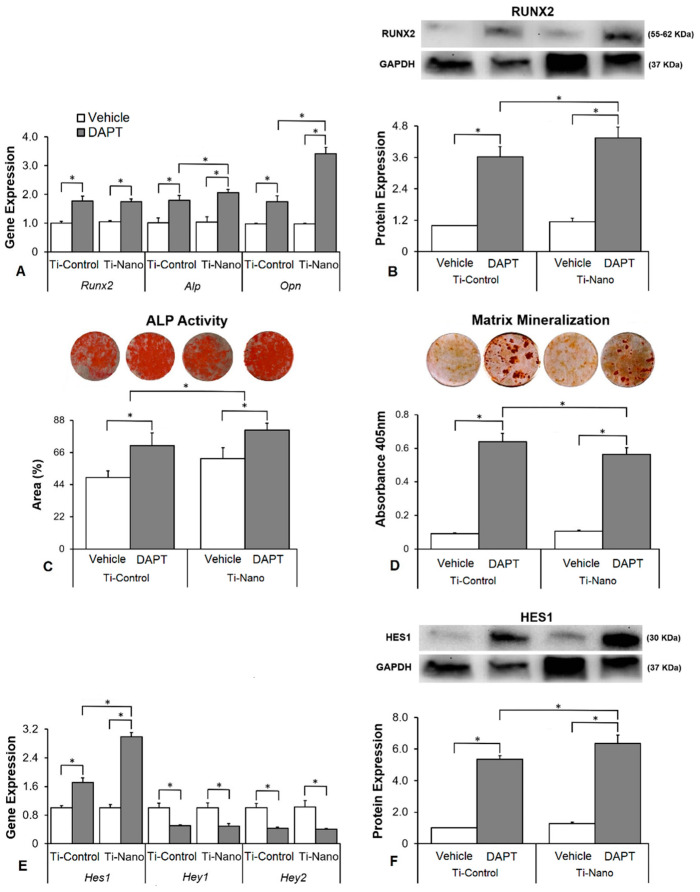
Effects of the Notch antagonist DAPT on osteoblast differentiation and the expression of Notch receptors in osteoblasts grown on polished Ti (Ti-Control) and Ti with nanotopography (Ti-Nano). The gene expression of the osteoblastic markers runt-related transcription factor 2 (*Runx2*), osteopontin (*Opn*), and alkaline phosphatase activity (*Alp*) on day 10 (**A**), RUNX2 protein expression on day 10 (**B**), ALP activity on day 7 (**C**), extracellular matrix mineralization on day 17 (**D**), gene expression of the Notch receptors hairy/enhancer of split 1 (*Hes1*) and hairy-related transcription factors 1 and 2 (*Hey1* and *Hey2*) on day 10 (**E**) and HES1 protein expression on day 10 (**F**) in osteoblasts cultured on Ti-Control and Ti-Nano with either vehicle or DAPT 20 μM. The original diameter of the Ti discs presented in C and D is 13 mm. The data of gene expression (n = 4), protein expression (n = 3), ALP activity (n = 5), and extracellular matrix mineralization (n = 5) are presented as the mean ± SD, and * indicates statistically significant differences (*p* ≤ 0.05).

The interaction between Ti surfaces and DAPT treatment did not affect the gene expression of *Runx2* (*p* = 0.757) but affected *Alp* (*p* = 0.043) and *Opn* (*p* = 0.026, Figure 5A). DAPT upregulated the gene expression of *Runx2* (*p* < 0.001 and *p* < 0.001), *Alp* (*p* < 0.001 and *p* = 0.008), and *Opn* (*p* < 0.001 and *p* < 0.001) in cells grown on both Ti-Control and Ti-Nano (Figure 5A). In the presence of vehicle, the gene expression of *Runx2* (*p* = 0.114), *Alp* (*p* = 0.863), and *Opn* (*p* = 0.938) was not affected by Ti surfaces (Figure 5A). In the presence of DAPT, the gene expression of *Runx2* (*p* = 0.233) were not affected, while *Alp* (*p* = 0.011) and *Opn* (*p* = 0.005) were higher in cells grown on Ti-Nano than on Ti-Control (Figure 5A). The interaction between Ti surfaces and DAPT treatment also affected the RUNX2 protein expression (*p* ≤ 0.001, Figure 5B). DAPT increased the RUNX2 protein expression (*p* < 0.001 and *p* < 0.001) in cells grown on both Ti-Control and Ti-Nano (Figure 5B). In the presence of vehicle, the RUNX2 protein expression was not affected (*p* = 0.571), while in the presence of DAPT, it was higher (*p* = 0.016) in cells grown on Ti-Nano than on Ti-Control (Figure 5B). The interaction between Ti surfaces and DAPT treatment affected the ALP activity (*p* ≤ 0.001, Figure 5C) and extracellular matrix mineralization (*p* = 0.014, Figure 5D). DAPT increased the ALP activity (*p* < 0.001 and *p* < 0.001, Figure 5C) and extracellular matrix mineralization (*p* < 0.001 and *p* < 0.001, Figure 5D) in cells grown on both Ti-Control and Ti-Nano. In the presence of either vehicle or DAPT, the ALP activity (*p* = 0.007 and *p* = 0.023) was greater in cells grown on Ti-Nano than on Ti-Control (Figure 5C). In the presence of vehicle, the extracellular matrix mineralization was not affected (*p* = 0.527), while in the presence of DAPT, it was greater (*p* = 0.005) in cells grown on Ti-Nano than on Ti-Control (Figure 5D). The interaction between Ti surfaces and DAPT treatment affected the gene expression of *Hes1* (*p* = 0.002) but not of *Hey1* (*p* = 0.869) and *Hey2* (*p* = 0.681, Figure 5E). DAPT upregulated the gene expression of *Hes1* (*p* < 0.001 and *p* < 0.001) and downregulated *Hey1* (*p* < 0.001 and *p* < 0.001), and *Hey2* (*p* < 0.001 and *p* < 0.001) in cells grown on both Ti-Control and Ti-Nano (Figure 5E). In the presence of vehicle, the gene expression of *Hes1* (*p* = 0.202), *Hey1* (*p* = 0.975), and *Hey2* (*p* = 0.771) was not affected by Ti surfaces (Figure 5E). In the presence of DAPT, the gene expression of *Hes1* (*p* = 0.001) was higher in cells grown on Ti-Nano than on Ti-Control, while *Hey1* (*p* = 0.840) and *Hey2* (*p* = 0.772) were not affected by Ti surfaces (Figure 3E). The interaction between Ti surfaces and DAPT treatment also affected the HES1 protein expression (*p* ≤ 0.001, Figure 5F). DAPT increased the HES1 protein expression (*p* < 0.001 and *p* < 0.001) in cells grown on both Ti-Control and Ti-Nano (Figure 5F). In the presence of vehicle, the HES1 protein expression was not affected (*p* = 0.274), while in the presence of DAPT, it was higher (*p* = 0.003) in cells grown on Ti-Nano than on Ti-Control (Figure 5F). 

**Figure 6:**
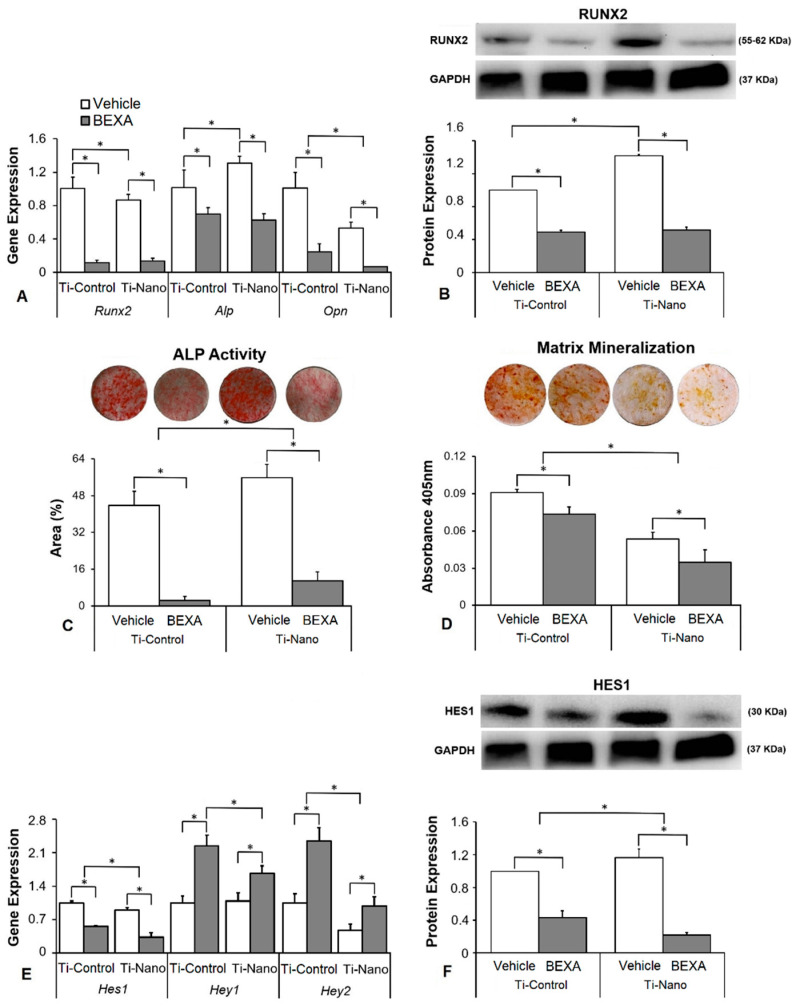
Effects of the Notch agonist bexarotene (BEXA) on osteoblast differentiation and the expression of Notch receptors in osteoblasts grown on polished Ti (Ti-Control) and Ti with nanotopography (Ti-Nano). The gene expression of the osteoblastic markers runt-related transcription factor 2 (*Runx2*), osteopontin (*Opn*), and alkaline phosphatase activity (*Alp*) on day 10 (**A**), RUNX2 protein expression on day 10 (**B**), ALP activity on day 7 (**C**), extracellular matrix mineralization on day 17 (**D**), gene expression of the Notch receptors hairy/enhancer of split 1 (*Hes1*) and hairy-related transcription factors 1 and 2 (*Hey1* and *Hey2*) on day 10 (**E**), and HES1 protein expression on day 10 (**F**), in osteoblasts cultured on Ti-Control and Ti-Nano with either vehicle or BEXA 0.1 μM. The original diameter of the Ti discs presented in C and D is 13 mm. The data of gene expression (n = 4), protein expression (n = 3), ALP activity (n = 5), and extracellular matrix mineralization (n = 5) are presented as the mean ± SD, and * indicates statistically significant differences (*p* ≤ 0.05).

The interaction between Ti surfaces and bexarotene treatment did not affect the gene expression of *Runx2* (*p* = 0.061) but affected *Alp* (*p* = 0.014) and *Opn* (*p* = 0.019, Figure 6A). Bexarotene downregulated the gene expression of *Runx2* (*p* < 0.001 and *p* < 0.001), *Alp* (*p* = 0.004 and *p* < 0.001), and *Opn* (*p* < 0.001 and *p* < 0.001) in cells grown on both Ti-Control and Ti-Nano (Figure 6A). In the presence of vehicle, the gene expression of *Runx2* (*p* = 0.024) and *Opn* (*p* < 0.001) was lower, while *Alp* (*p* = 0.007) and was higher in cells grown on Ti-Nano than on Ti-Control (Figure 6A). In the presence of bexarotene, the gene expression of *Runx2* (*p* = 0.746) and *Alp* (*p* = 0.439) was not affected, while *Opn* (*p* = 0.041) was lower in cells grown on Ti-Nano than on Ti-Control (Figure 6A). The interaction between Ti surfaces and bexarotene treatment also affected the RUNX2 protein expression (*p* ≤ 0.001, Figure 6B). Bexarotene decreased the RUNX2 protein expression (*p* < 0.001 and *p* < 0.001) in cells grown on both Ti-Control and Ti-Nano (Figure 6B). In the presence of vehicle, the RUNX2 protein expression was higher (*p* < 0.001) in cells grown on Ti-Nano than on Ti-Control, while in the presence of bexarotene, it was not affected (*p* = 0.731) by Ti surfaces (Figure 6B). The interaction between Ti surfaces and bexarotene treatment affected the ALP activity (*p* ≤ 0.001, Figure 6C) and extracellular matrix mineralization (*p* ≤ 0.001, Figure 6D). Bexarotene decreased the ALP activity (*p* < 0.001 and *p* < 0.001, Figure 6C) and extracellular matrix mineralization (*p* = 0.002 and *p* = 0.001, Figure 6D) in cells grown on both Ti-Control and Ti-Nano. In the presence of vehicle or bexarotene, the ALP activity (*p* = 0.001 and *p* = 0.011) was higher in cells grown on Ti-Nano than on Ti-Control (Figure 6C). In the presence of vehicle or bexarotene, the extracellular matrix mineralization (*p* < 0.001 and *p* < 0.001) was lower in cells grown on Ti-Nano than on Ti-Control (Figure 6D). The interaction between Ti surfaces and bexarotene treatment affected the gene expression of *Hes1* (*p* ≤ 0.001), *Hey1* (*p* = 0.004), and *Hey2* (*p* = 0.003, Figure 6E). Bexarotene downregulated the gene expression of *Hes1* (*p* < 0.001 and *p* < 0.001) and upregulated *Hey1* (*p* < 0.001 and *p* < 0.001) and *Hey2* (*p* < 0.001 and *p* = 0.004) in cells grown on both Ti-Control and Ti-Nano (Figure 6E). In the presence of vehicle, the gene expression *Hey1* (*p* = 0.740) was not affected, while *Hes1* (*p* = 0.004) and *Hey2* (*p* = 0.002) were higher in cells grown on Ti-Control than on Ti-Nano (Figure 6E). In the presence of bexarotene, the gene expression of *Hes1* (*p* < 0.001), *Hey1* (*p* < 0.001), and *Hey2* (*p* < 0.001) was lower in cells grown on Ti-Nano than on Ti-Control (Figure 6E). The interaction between Ti surfaces and bexarotene treatment also affected the HES1 protein expression (*p* = 0.001, Figure 6F). Bexarotene decreased the HES1 protein expression (*p* < 0.001 and *p* < 0.001) in cells grown on both Ti-Control and Ti-Nano (Figure 4F). In the presence of vehicle, the HES1 protein expression was higher (*p* = 0.021), while, in the presence of bexarotene, it was lower (*p* = 0.005) in cells grown on Ti-Nano than on Ti-Control (Figure 6F).

### 3.5. Effects of the Combination of the Hedgehog and Notch Signalling Modulation on the Gene Expression of Bone Markers in the Cells Grown on Ti-Control and Ti-Nano

**Figure 7:**
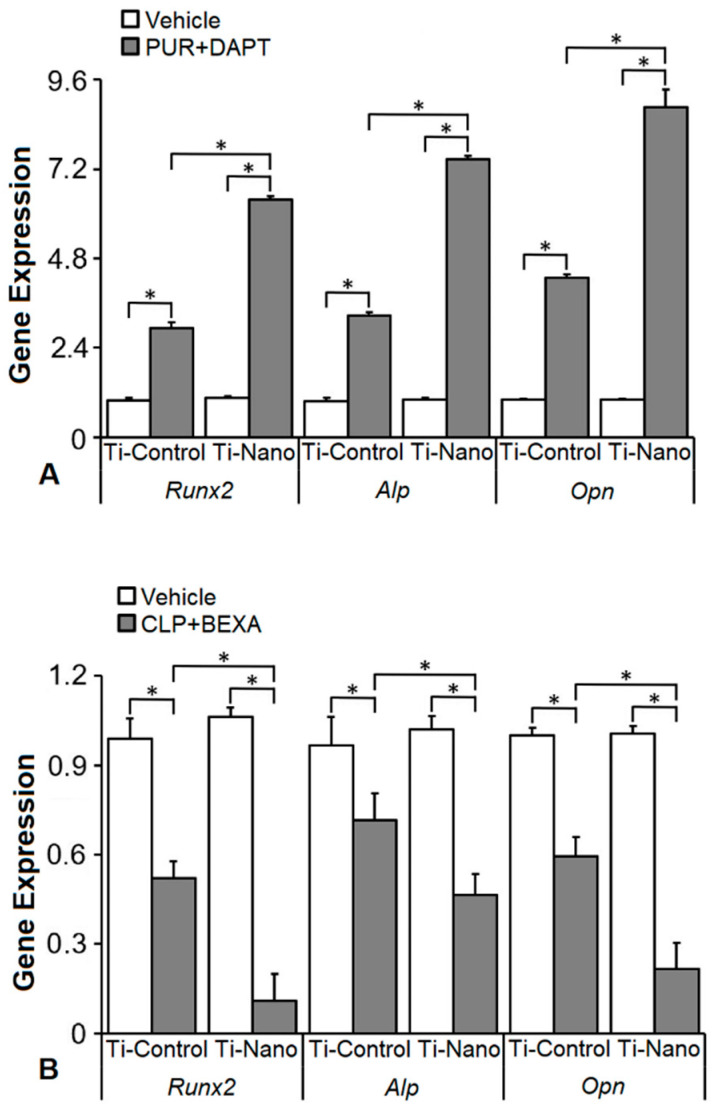
Effects of the combination of the Hedgehog agonist purmorphamine (PUR) with Notch antagonist DAPT and Hedgehog antagonist cyclopamine (CLP) with Notch agonist bexarotene (BEXA) on the gene expression of bone markers in osteoblasts grown on polished Ti (Ti-Control) and Ti with nanotopography (Ti-Nano). The gene expression of runt-related transcription factor 2 (*Runx2*), osteopontin (*Opn*), and alkaline phosphatase activity (*Alp*) on day 10 in osteoblasts cultured on Ti-Control and Ti-Nano with either vehicle or the combination of purmorphamine 2 μM with DAPT 20 μM (PUR+DAPT, (**A**)) or with either vehicle or the combination of cyclopamine 10 nM with bexarotene 0.1 μM (CLP+BEXA) (**B**). The data (n = 4) are presented as the mean ± SD, and * indicates statistically significant differences (*p* ≤ 0.05).

The interaction between Ti surfaces and the treatment with the combination of purmorphamine and DAPT affected the expression of *Runx2* (*p* ≤ 0.001), *Alp* (*p* ≤ 0.001), and *Opn* (*p* ≤ 0.001, Figure 7). The combination of purmorphamine and DAPT upregulated the gene expression of *Runx2* (*p* < 0.001 and *p* < 0.001), *Alp* (*p* < 0.001 and *p* < 0.001), and *Opn* (*p* < 0.001 and *p* < 0.001) in cells grown on both Ti-Control and Ti-Nano (Figure 7A). In the presence of vehicle, the gene expression of *Runx2* (*p* = 0.707), *Alp* (*p* = 0.345), and *Opn* (*p* = 0.969) was not affected by Ti surfaces (Figure 7A). In the presence of the combination of purmorphamine and DAPT, the gene expression of *Runx2* (*p* < 0.001), *Alp* (*p* < 0.001), and *Opn* (*p* < 0.001) was higher in cells grown on Ti-Nano than on Ti-Control (Figure 7A). The interaction between Ti surfaces and the treatment with the combination of cyclopamine and bexarotene affected the expression of *Runx2* (*p* ≤ 0.001), *Alp* (*p* = 0.010), and *Opn* (*p* ≤ 0.001, Figure 7B). The combination of cyclopamine and bexarotene downregulated the gene expression of *Runx2* (*p* = 0.001 and *p* < 0.001), *Alp* (*p* = 0.001 and *p* < 0.001), and *Opn* (*p* = 0.001 and *p* < 0.001) in cells grown on both Ti-Control and Ti-Nano (Figure 7B). In the presence of vehicle, the gene expression of *Runx2* (*p* = 0.248), *Alp* (*p* = 0.416), and *Opn* (*p* = 0.874) were not affected by Ti surfaces (Figure 7B). In the presence of the combination of cyclopamine and bexarotene, the gene expression of *Runx2* (*p* < 0.001), *Alp* (*p* = 0.005), and *Opn* (*p* < 0.001) was lower in cells grown on Ti-Nano than on Ti-Control (Figure 7B).

## 4. Discussion

The modulation of the cell signalling involved in osteogenesis impacts the interaction between osteoblasts and Ti surfaces [40,42,45,53,54]. This study showed that agonists and antagonists of the Hedgehog and Notch signalling pathways affect osteoblast differentiation. Using either the Hedgehog agonist purmorphamine or the Notch antagonist DAPT increased while the Hedgehog antagonist cyclopamine or the Notch agonist bexarotene decreased the osteoblast differentiation of cells cultured on Ti-Control and Ti-Nano. Additionally, the association between purmorphamine and DAPT seems to have a synergistic effect in increasing the osteoblast differentiation of cells grown on both Ti surfaces, especially on Ti-Nano.

To select the concentration of the Hedgehog and Notch agonists and antagonists, we tested three doses of each, based on data from the literature, and evaluated three critical parameters of osteoblast differentiation; *Opn* gene expression, ALP activity, and extracellular matrix mineralization [18,19,32,34]. As observed in other studies, the Hedgehog agonist purmorphamine was more efficient in inducing osteoblast differentiation at a concentration of 2 μM [14,15,55]. The Hedgehog antagonist cyclopamine inhibited the osteoblast differentiation and because the concentration of 1000 nM seems to induce some toxicity specifically based on its effect on *Opn* gene expression and ALP activity, we selected 10 nM as it was more efficient than 100 nM in inhibiting osteoblast differentiation [56,57,58]. In agreement with previous studies, the Notch antagonist DAPT at the concentration of 20 µM was more osteogenic [33,59,60]. The Notch agonist bexarotene was more efficient in reducing osteoblast differentiation at the concentration of 0.1 µM, which agrees with previous studies [61,62,63].

Hedgehog agonist purmorphamine being used to enhance the osteoblast differentiation of cells grown on Ti has already been investigated [15,64]. Herein, we showed that the osteogenic effects of purmorphamine were more prominent on Ti-Nano, as evidenced by an increase in gene expression of *Runx2*, *Opn*, and *Alp*, RUNX2 protein expression, and ALP activity, which were more evident in the cells grown on Ti-Nano than on Ti-Control. The Hedgehog antagonist cyclopamine inhibited the osteoblast differentiation in a more pronounced way in cells grown on Ti-Nano compared with Ti-Control, as shown by the gene expression of *Runx2*, *Opn*, and *Alp*, and RUNX2 protein expression. This higher susceptibility to the Hedgehog agonist and antagonist of cells grown on Ti-Nano in osteoblast differentiation could be related to the modulation of the Hedgehog signalling pathway being more intense in cells grown on this surface. Indeed, the gene expression of the Hedgehog receptors *Gli1*, *Gli2*, and *Gli3*, and the GLI1 protein expression were more modulated by purmorphamine and cyclopamine except for GLI1 protein expression, with the expected opposite effects of the agonist and antagonist. Together, these results suggest that the Hedgehog signalling pathway is more relevant to the osteogenic potential of the Ti-Nano than of Ti-Control and that this nanotopography can regulate this cellular mechanism by itself. Despite few information on this subject is available in the literature, it was demonstrated that Ti with micro-/nanotextured topography, either with or without TiO_2_ nanotubes, enhances osteoblast differentiation of MG63 cell lineage by activating Hedgehog-Gli1 signalling, which is inhibited by cyclopamine [65].

The involvement of the Notch signalling pathway in the osteoblast-Ti interaction is underexplored, despite its well-known participation in osteogenesis [31,66,67]. The Ti surface hydrophilicity was observed to favour bone formation by acting on several signalling pathways involved in proliferation and osteoblast precursor differentiation, including Notch signalling [68]. Additionally, the inhibition of the Notch signalling enhances the osteoblast differentiation of mesenchymal stem cells cultured on Ti substrates [69]. In keeping with this, we demonstrated that the Notch antagonist DAPT enhanced the osteoblast differentiation of cells grown on both Ti-Control and Ti-Nano, with more pronounced effects on Ti-Nano as noticed by the gene expression of *Opn* and *Alp*, RUNX2 protein expression, and ALP activity. Corroborating these data, the Notch agonist bexarotene inhibited the osteoblast differentiation more intensely in the cells grown on Ti-Nano than on Ti-Control, by reducing the same parameters and the extracellular matrix mineralization. As for the Hedgehog signalling, the higher responsiveness to the Notch antagonist and agonist of cells grown on Ti-Nano regarding osteoblast differentiation could be attributed to the higher intensity of the regulation of the Notch signalling in cells grown on this surface, specifically through the regulation of the Notch receptor Hes1. Indeed, the gene and protein expression of Hes1 was more modulated by DAPT and Bexarotene in cells grown on Ti-Nano than on Ti-Control while the gene expression of *Hey1* and *Hey2* was not affected by surface topography. Collectively, these data suggest that the Notch signalling pathway is more important to the osteogenic potential of the Ti-Nano than Ti-Control and that this nanotopography can regulate this signal. Although the participation of the Notch signalling in the osteoblast differentiation of cells grown on Ti surfaces has already been described, to the best of our knowledge, this is the first evidence that the distinct effects elicited by different Ti surface topographies on osteoblast differentiation involve the regulation of this signalling pathway [68,69].

As our results showed more prominent effects of the modulation of the Hedgehog and Notch signalling in osteoblasts grown on Ti-Nano, we started an investigation on the possible synergistic effect of the combination of the modulation of both signalling pathways. Although the effect of this combination on osteoblast differentiation was not previously evaluated, the only study presented in the literature is not related to bone tissue and demonstrated that the concomitant regulation of the Hedgehog and Notch signalling pathways potentiates the anti-leukemic effects of the Notch modulation alone [70]. Here, the association of the Hedgehog agonist purmorphamine with the Notch antagonist DAPT increased the upregulation of the *Runx2*, *Opn*, and *Alp* gene expression compared with the use of either purmorphamine or DAPT alone in osteoblasts grown on both Ti surfaces with more intense effects on Ti-Nano compared with Ti-Control. Although combining the Hedgehog antagonist cyclopamine with the Notch agonist bexarotene downregulated these gene expressions, the synergistic effect was not as evident as we observed when purmorphamine and DAPT were combined. Thus, despite further studies are needed to confirm the synergism, it is possible to suggest that the activation of Hedgehog along with the inhibition of Notch signalling may favour the osteoblast differentiation of cells grown on Ti, especially with nanostructured surfaces.

In conclusion, our results indicate that the Hedgehog and Notch signalling pathways are involved in the responses of osteoblasts to Ti surfaces, with more relevant effects on osteoblast differentiation of cells grown on the nanostructured surface, which may regulate these signals by itself. We also demonstrated that the concomitant activation of Hedgehog and inhibition of Notch might synergistically affect osteoblast differentiation, especially in cells grown on nanotopography. These cellular mechanisms may explain, at least in part, the higher osteogenic potential of this nanostructured Ti surface, which opens windows to develop strategies to drive the process of osseointegration.

## Figures and Tables

**Table 1:** Primer sequences for RT-qPCR.

Gene	Forward	Reverse
*Opn*	GAAGCCTGACCCATCTCAGAA	GTTGCTTGGAAGAGTTTCTTGCTT
*Runx2*	CGTATTTCAGATGATGACACTGCC	AAATGCCTGGGAACTGCCTG
*Alp*	TACTGCTGATCACTCCCACG	ACCGTCCACCACCTTGTAAC
*Gli1*	ACCTGCAAACCGTAATCCGT	TCCTAAAGAAGGGCTCATGGTG
*Gli2*	CCAACCAGAATAAGCAGAACAGC	TGAGATCAGCCAGTTGCTCC
*Gli3*	AGTCAGCCCTGCGGAATACT	GGGAAATCTGGTGCTGTCCAT
*Hes1*	ACGACACCGGACAAACCAAA	CGGGAGCTATCTTTCTTAAGTGCAT
*Hey1*	GCCGACGAGACCGAATCAAT	ATAGTCCATAGCCAGGGCGT
*Hey2*	CGTGGGGAGCGAGAACAATTA	ATTTATTCGATCCCGACGCCT
*Eif2β*	ACCTCCCTGGAATACTCTGACT	TCGCCCCGTCTTTGATGAAT

## Data Availability

The datasets used and/or analysed during the current study are available from the corresponding author on reasonable request.

## References

[1] Adolpho L.F., Lopes H.B., Freitas G.P., Weffort D., Campos Totoli G.G., Loyola Barbosa A.C., Freire Assis R.I., Silverio Ruiz K.G., Andia D.C., Rosa A.L. (2022). Human periodontal ligament stem cells with distinct osteogenic potential induce bone formation in rat calvaria defects. Regen. Med..

[2] Chen Q., Liu W., Sinha K.M., Yasuda H., de Crombrugghe B. (2013). Identification and characterization of microRNAs controlled by the osteoblast-specific transcription factor Osterix. PLoS ONE.

[3] Souza A.T.P., Lopes H.B., Oliveira F.S., Weffort D., Freitas G.P., Adolpho L.F., Fernandes R.R., Rosa A.L., Beloti M.M. (2021). The extracellular matrix protein Agrin is expressed by osteoblasts and contributes to their differentiation. Cell Tissue Res..

[4] Yang J., Andre P., Ye L., Yang Y.-Z. (2015). The Hedgehog signalling pathway in bone formation. Int. J. Oral Sci..

[5] Levi B., James A.W., Nelson E.R., Li S., Peng M., Commons G.W., Lee M., Wu B., Longaker M.T. (2011). Human Adipose-Derived Stromal Cells Stimulate Autogenous Skeletal Repair via Paracrine Hedgehog Signaling with Calvarial Osteoblasts. Stem Cells Dev..

[6] Yan X., Yang Z., Chen Y., Li N., Wang L., Dou G., Liu Y., Duan J., Feng L., Deng S. (2015). Endothelial cells-targeted soluble human Delta-like 4 suppresses both physiological and pathological ocular angiogenesis. Sci. China Life Sci..

[7] Heretsch P., Tzagkaroulaki L., Giannis A. (2010). Cyclopamine and Hedgehog Signaling: Chemistry, Biology, Medical Perspectives. Angew. Chem. Int. Ed..

[8] Roberge L., Origa-Alves A.C., Rebelatto C.L.K., Dallagiovanna B., Shigunov P. (2013). Inhibition of Hedgehog signaling pathway affects the expression of miR-20a and miR-3. J. Biotechnol. Biodivers..

[9] Ullah A., Ullah N., Nawaz T., Aziz T. (2022). Molecular mechanisms of Sanguinarine in cancer prevention and treatment. Anti-Cancer Agents Med. Chem..

[10] Wang Q., Huang C., Zeng F., Xue M., Zhang X. (2010). Activation of the Hh pathway in periosteum-derived mesenchymal stem cells induces bone formation in vivo: Implication for postnatal bone repair. Am. J. Pathol..

[11] Evangelista M., Tian H., de Sauvage F.J. (2006). The hedgehog signaling pathway in cancer. Clin. Cancer Res. Off. J. Am. Assoc. Cancer Res..

[12] Ingham P.W., McMahon A.P. (2001). Hedgehog signaling in animal development: Paradigms and principles. Genes Dev..

[13] Plaisant M., Giorgetti-Peraldi S., Gabrielson M., Loubat A., Dani C., Peraldi P. (2011). Inhibition of hedgehog signaling decreases proliferation and clonogenicity of human mesenchymal stem cells. PLoS ONE.

[14] Beloti M.M., Bellesini L.S., Rosa A.L. (2005). Purmorphamine enhances osteogenic activity of human osteoblasts derived from bone marrow mesenchymal cells. Cell Biol. Int..

[15] Beloti M.M., Bellesini L.S., Rosa A.L. (2005). The effect of purmorphamine on osteoblast phenotype expression of human bone marrow mesenchymal cells cultured on titanium. Biomaterials.

[16] Oliveira F.S., Bellesini L.S., Defino H.L.A., da Silva Herrero C.F., Beloti M.M., Rosa A.L. (2012). Hedgehog signaling and osteoblast gene expression are regulated by purmorphamine in human mesenchymal stem cells. J. Cell. Biochem..

[17] Wu X., Ding S., Ding Q., Gray N.S., Schultz P.G. (2002). A small molecule with osteogenesis-inducing activity in multipotent mesenchymal progenitor cells. J. Am. Chem. Soc..

[18] Sinha S., Chen J.K. (2006). Purmorphamine activates the Hedgehog pathway by targeting Smoothened. Nat. Chem. Biol..

[19] Wu X., Walker J., Zhang J., Ding S., Schultz P.G. (2004). Purmorphamine induces osteogenesis by activation of the hedgehog signaling pathway. Chem. Biol..

[20] Heretsch P., Tzagkaroulaki L., Giannis A. (2010). Modulators of the hedgehog signaling pathway. Bioorganic Med. Chem..

[21] Katoh Y., Katoh M. (2009). Hedgehog target genes: Mechanisms of carcinogenesis induced by aberrant hedgehog signaling activation. Curr. Mol. Med..

[22] Quint E., Smith A., Avaron F., Laforest L., Miles J., Gaffield W., Akimenko M.-A. (2002). Bone patterning is altered in the regenerating zebrafish caudal fin after ectopic expression of sonic hedgehog and bmp2b or exposure to cyclopamine. Proc. Natl. Acad. Sci. USA.

[23] Suh J.M., Gao X., McKay J., McKay R., Salo Z., Graff J.M. (2006). Hedgehog signaling plays a conserved role in inhibiting fat formation. Cell Metab..

[24] Panepucci R.A., Oliveira L.H.B., Zanette D.L., Viu Carrara R.d.C., Araujo A.G., Orellana M.D., Bonini de Palma P.V., Menezes C.C.B.O., Covas D.T., Zago M.A. (2010). Increased levels of NOTCH1, NF-kappaB, and other interconnected transcription factors characterize primitive sets of hematopoietic stem cells. Stem Cells Dev..

[25] Radtke F., Schweisguth F., Pear W. (2005). The Notch “gospel”. EMBO Rep..

[26] High F., Epstein J.A. (2007). Signalling Pathways Regulating Cardiac Neural Crest Migration and Differentiation. Vascular Development.

[27] Fischer A., Gessler M. (2007). Delta-Notch—And then? Protein interactions and proposed modes of repression by Hes and Hey bHLH factors. Nucleic Acids Res..

[28] Garg V., Muth A.N., Ransom J.F., Schluterman M.K., Barnes R., King I.N., Grossfeld P.D., Srivastava D. (2005). Mutations in NOTCH1 cause aortic valve disease. Nature.

[29] McLarren K.W., Lo R., Grbavec D., Thirunavukkarasu K., Karsenty G., Stifani S. (2000). The mammalian basic helix loop helix protein HES-1 binds to and modulates the transactivating function of the runt-related factor Cbfa1. J. Biol. Chem..

[30] Shen Q., Christakos S. (2005). The vitamin D receptor, Runx2, and the Notch signaling pathway cooperate in the transcriptional regulation of osteopontin. J. Biol. Chem..

[31] Zamurovic N., Cappellen D., Rohner D., Susa M. (2004). Coordinated activation of notch, Wnt, and transforming growth factor-beta signaling pathways in bone morphogenic protein 2-induced osteogenesis. Notch target gene Hey1 inhibits mineralization and Runx2 transcriptional activity. J. Biol. Chem..

[32] Chitranshi N., Dheer Y., Kumar S., Graham S.L., Gupta V. (2019). Molecular docking, dynamics, and pharmacology studies on bexarotene as an agonist of ligand-activated transcription factors, retinoid X receptors. J. Cell. Biochem..

[33] Dishowitz M.I., Terkhorn S.P., Bostic S.A., Hankenson K.D. (2012). Notch signaling components are upregulated during both endochondral and intramembranous bone regeneration. J. Orthop. Res..

[34] Jing W., Xiong Z., Cai X., Huang Y., Li X., Yang X., Liu L., Tang W., Lin Y., Tian W. (2010). Effects of γ-secretase inhibition on the proliferation and vitamin D3 induced osteogenesis in adipose derived stem cells. Biochem. Biophys. Res. Commun..

[35] Costa D.G., Ferraz E.P., Abuna R.P.F., de Oliveira P.T., Morra M., Beloti M.M., Rosa A.L. (2017). The effect of collagen coating on titanium with nanotopography on in vitro osteogenesis. J. Biomed. Mater. Res. A.

[36] Martin J.Y., Schwartz Z., Hummert T.W., Schraub D.M., Simpson J., Lankford J., Dean D.D., Cochran D.L., Boyan B.D. (1995). Effect of titanium surface roughness on proliferation, differentiation, and protein synthesis of human osteoblast-like cells (MG63). J. Biomed. Mater. Res..

[37] Mendonça G., Mendonça D.B.S., Simões L.G.P., Araújo A.L., Golin A.L., Duarte W.R., Cooper L.F., Aragão F.J.L. (2010). Efeito de superfícies de implantes nano-estruturadas na expressão de genes de osteoblastos e no contato osso-implante in vivo. Rev. Odontológica Bras. Cent..

[38] Silverwood R.K., Fairhurst P.G., Sjöström T., Welsh F., Sun Y., Li G., Yu B., Young P.S., Su B., Meek R.M.D. (2016). Analysis of Osteoclastogenesis/Osteoblastogenesis on Nanotopographical Titania Surfaces. Adv. Healthc. Mater..

[39] Rosa A., Kato R., Castro-Raucci L., Teixeira L., de Oliveira F., Bellesini L., de Oliveira P.T., Hassan M., Beloti M. (2014). Nanotopography drives stem cell fate toward osteoblast differentiation through α1β1 integrin signaling pathway. J. Cell. Biochem..

[40] Bighetti-Trevisan R.L., Almeida L.O., Castro-Raucci L.M.S., Gordon J.A.R., Tye C.E., Stein G.S., Lian J.B., Stein J.L., Rosa A.L., Beloti M.M. (2022). Titanium with nanotopography attenuates the osteoclast-induced disruption of osteoblast differentiation by regulating histone methylation. Biomater. Adv..

[41] de Oliveira P.T., Zalzal S.F., Beloti M.M., Rosa A.L., Nanci A. (2007). Enhancement of in vitro osteogenesis on titanium by chemically produced nanotopography. J. Biomed. Mater. Res. A.

[42] Abuna R.P.F., Oliveira F.S., Adolpho L.F., Fernandes R.R., Rosa A.L., Beloti M.M. (2020). Frizzled 6 disruption suppresses osteoblast differentiation induced by nanotopography through the canonical Wnt signaling pathway. J. Cell. Physiol..

[43] Kato R.B., Roy B., De Oliveira F.S., Ferraz E.P., De Oliveira P.T., Kemper A.G., Hassan M.Q., Rosa A.L., Beloti M.M. (2014). Nanotopography Directs Mesenchymal Stem Cells to Osteoblast Lineage through Regulation of microRNA-SMAD-BMP-2 Circuit. J. Cell. Physiol..

[44] Lopes H.B., Freitas G.P., Elias C.N., Tye C., Stein J.L., Stein G.S., Lian J.B., Rosa A.L., Beloti M.M. (2019). Participation of integrin β3 in osteoblast differentiation induced by titanium with nano or microtopography. J. Biomed. Mater. Res. A.

[45] Lopes H.B., Souza A.T.P., Freitas G.P., Elias C.N., Rosa A.L., Beloti M.M. (2020). Effect of focal adhesion kinase inhibition on osteoblastic cells grown on titanium with different topographies. J. Appl. Oral Sci. Rev. FOB.

[46] Castro-Raucci L.M.S., Francischini M.S., Teixeira L.N., Ferraz E.P., Lopes H.B., de Oliveira P.T., Hassan M.Q., Rosa A.L., Beloti M.M. (2016). Titanium With Nanotopography Induces Osteoblast Differentiation by Regulating Endogenous Bone Morphogenetic Protein Expression and Signaling Pathway. J. Cell. Biochem..

[47] de Oliveira P.T., Zalzal S.F., Irie K., Nanci A. (2003). Early Expression of Bone Matrix Proteins in Osteogenic Cell Cultures. J. Histochem. Cytochem..

[48] Tambasco de Oliveira P., Nanci A. (2004). Nanotexturing of titanium-based surfaces upregulates expression of bone sialoprotein and osteopontin by cultured osteogenic cells. Biomaterials.

[49] Abuna R.P.F., Oliveira F.S., Ramos J.I.R., Lopes H.B., Freitas G.P., Souza A.T.P., Beloti M.M., Rosa A.L. (2018). Selection of reference genes for quantitative real-time polymerase chain reaction studies in rat osteoblasts. J. Cell. Physiol..

[50] Livak K.J., Schmittgen T.D. (2001). Analysis of relative gene expression data using real-time quantitative PCR and the 2(-Delta Delta C(T)) Method. Methods.

[51] Majors A.K., Boehm C.A., Nitto H., Midura R.J., Muschler G.F. (1997). Characterization of human bone marrow stromal cells with respect to osteoblastic differentiation. J. Orthop. Res..

[52] Gregory C.A., Gunn W.G., Peister A., Prockop D.J. (2004). An Alizarin red-based assay of mineralization by adherent cells in culture: Comparison with cetylpyridinium chloride extraction. Anal. Biochem..

[53] Boyan B.D., Berger M.B., Nelson F.R., Donahue H.J., Schwartz Z. (2022). The Biological Basis for Surface-dependent Regulation of Osteogenesis and Implant Osseointegration. JAAOS-J. Am. Acad. Orthop. Surg..

[54] Zhao Y., Bai L., Zhang Y., Yao R., Sun Y., Hang R., Chen X., Wang H., Yao X., Xiao Y. (2022). Type I collagen decorated nanoporous network on titanium implant surface promotes osseointegration through mediating immunomodulation, angiogenesis, and osteogenesis. Biomaterials.

[55] van der Horst G., Farih-Sips H., Löwik C.W.G.M., Karperien M. (2003). Hedgehog stimulates only osteoblastic differentiation of undifferentiated KS483 cells. Bone.

[56] Chen J.K., Taipale J., Cooper M.K., Beachy P.A. (2002). Inhibition of Hedgehog signaling by direct binding of cyclopamine to Smoothened. Genes Dev..

[57] Stanton B.Z., Peng L.F. (2009). Small-molecule modulators of the Sonic Hedgehog signaling pathway. Mol. Biosyst..

[58] Yuan Y.-F., Zhu W.-X., Liu T., He J.-Q., Zhou Q., Zhou X., Zhang X., Yang J. (2020). Cyclopamine functions as a suppressor of benign prostatic hyperplasia by inhibiting epithelial and stromal cell proliferation via suppression of the Hedgehog signaling pathway. Int. J. Mol. Med..

[59] Androutsellis-Theotokis A., Leker R.R., Soldner F., Hoeppner D.J., Ravin R., Poser S.W., Rueger M.A., Bae S.-K., Kittappa R., McKay R.D.G. (2006). Notch signalling regulates stem cell numbers in vitro and in vivo. Nature.

[60] Canalis E. (2008). Notch Signaling in Osteoblasts. Sci. Signal..

[61] Ai X., Mao F., Shen S., Shentu Y., Wang J., Lu S. (2018). Bexarotene inhibits the viability of non-small cell lung cancer cells via slc10a2/PPARγ/PTEN/mTOR signaling pathway. BMC Cancer.

[62] Fantini J., Di Scala C., Yahi N., Troadec J.-D., Sadelli K., Chahinian H., Garmy N. (2014). Bexarotene blocks calcium-permeable ion channels formed by neurotoxic Alzheimer’s β-amyloid peptides. ACS Chem. Neurosci..

[63] Kamp F., Scheidt H.A., Winkler E., Basset G., Heinel H., Hutchison J.M., LaPointe L.M., Sanders C.R., Steiner H., Huster D. (2018). Bexarotene Binds to the Amyloid Precursor Protein Transmembrane Domain, Alters Its α-Helical Conformation, and Inhibits γ-Secretase Nonselectively in Liposomes. ACS Chem. Neurosci..

[64] Lin Y., Huang Y., He J., Chen F., He Y., Zhang W. (2017). Role of Hedgehog-Gli1 signaling in the enhanced proliferation and differentiation of MG63 cells enabled by hierarchical micro-/nanotextured topography. Int. J. Nanomed..

[65] Xie Y., Chen X., Zheng X., Li L., Li J., Xu Y., He J., Lin Y. (2021). Beta1-integrin/Hedgehog-Gli1 signaling pathway fuels the diameter-dependent osteoblast differentiation on different TiO2 nanotubes: The optimal-diameter nanotubes for osteoblast differentiation. Int. J. Biochem. Cell Biol..

[66] Delgado-Calle J., McAndrews K., Wu G., Orr A.L., Ferrari A., Tu X., Srinivasan V., Roodman G.D., Ebetino F.H., Boeckman R.K. (2022). The Notch pathway regulates the bone gain induced by PTH anabolic signaling. FASEB J..

[67] Zanotti S., Smerdel-Ramoya A., Stadmeyer L., Durant D., Radtke F., Canalis E. (2008). Notch inhibits osteoblast differentiation and causes osteopenia. Endocrinology.

[68] Calciolari E., Hamlet S., Ivanovski S., Donos N. (2018). Pro-osteogenic properties of hydrophilic and hydrophobic titanium surfaces: Crosstalk between signalling pathways in in vivo models. J. Periodontal Res..

[69] Wang H., Jiang Z., Zhang J., Xie Z., Wang Y., Yang G. (2017). Enhanced osteogenic differentiation of rat bone marrow mesenchymal stem cells on titanium substrates by inhibiting Notch3. Arch. Oral Biol..

[70] Okuhashi Y., Itoh M., Nara N., Tohda S. (2011). Effects of combination of notch inhibitor plus hedgehog inhibitor or Wnt inhibitor on growth of leukemia cells. Anti-Cancer Res..

